# Using social and sexual networking mobile applications to promote HIV testing, medical care and prevention services among Latino men who have sex with men in Los Angeles County, California, USA

**DOI:** 10.1371/journal.pone.0268406

**Published:** 2022-05-13

**Authors:** Frank H. Galvan, Honghu Liu, Ronald A. Brooks, Ying-Tung Chen, Ricardo Mendoza Lepe

**Affiliations:** 1 Department of Research and Evaluation, Bienestar Human Services, Inc., Los Angeles, California, United States of America; 2 Division of Public Health & Community Dentistry, School of Dentistry, University of California, Los Angeles, California, United States of America; 3 Division of General Internal Medicine & Health Services Research, David Geffen School of Medicine, University of California, Los Angeles, California, United States of America; 4 Department of Biostatistics, Jonathan and Karin Fielding School of Public Health, University of California, Los Angeles, California, United States of America; 5 Department of Family Medicine, David Geffen School of Medicine, University of California, Los Angeles, California, United States of America; UNSW Australia, AUSTRALIA

## Abstract

HIV disproportionately affects Latinos versus Whites, with Latinos having higher rates of HIV. Additionally, many HIV-positive Latinos are unaware of their infection. *Proyecto Protégete*, an HIV prevention intervention developed for Latino men who have sex with men (MSM), used social and sexual networking mobile applications (apps) to recruit individuals for HIV testing and linkage to medical care and prevention services. This study occurred in Los Angeles County, California, USA from December 18, 2015 to April 22, 2017. The study’s primary aim was to assess *Proyecto Protégete*‘s ability to successfully recruit Latino MSM involved in high-risk sexual activities. A secondary aim was to evaluate its capacity to promote HIV testing and linkage to HIV medical care and prevention services among this population by comparing it to two programs with similar samples. Comparisons using Fisher’s Exact Test were conducted between *Proyecto Protégete* and the HIV testing program of the agency in which *Proyecto Protégete* was located and the County-funded HIV testing programs to compare the samples’ rates of HIV-positive Latino MSM identified through HIV testing and linked to HIV medical care and prevention services. Participants were recruited through seven apps. In *Proyecto Protégete*, 9,573 individuals completed the screener, 4,657 were eligible, and 359 (7.7% of those eligible) enrolled. Among those enrolled, 79% reported anal sex without a condom in the previous twelve months; 51% reported anal sex under the influence of alcohol. The HIV positivity rates were as follows: *Proyecto Protégete*, 1.71%; the agency, 1.25% (*p* = .293, compared to *Proyecto Protégete*); and the County, 1.09% (*p* = .172, compared to *Proyecto Protégete*). The rates of those confirmed as new HIV-positives and linked to medical care within 30 days were as follows: *Proyecto Protégete*, 71.4%; the agency, 81.5% (*p* = .450, compared to *Proyecto Protégete*); and the County, 77.3% (*p* = .503, compared to *Proyecto Protégete*). *Proyecto Protégete* had a higher rate of linked referrals to prevention services than the agency’s testing program (19.5% versus 8.3%, *p* < .001). *Proyecto Protégete* experienced successes in some areas but not in others. Future research should build on *Proyecto Protégete*’s experiences to promote HIV-related services among Latino MSM.

## Introduction

HIV disproportionately affects Latinos compared to Whites. In 2019, according to the Centers for Disease Control and Prevention (CDC), the rate of HIV infection per 100,000 for Latinos was 16.7 compared to 4.6 for Whites [1]. Among HIV-positive Latinos, an estimated 16% were unaware of their infection [2]. In addition, for every 100 Latinos living with HIV, only 74 received some HIV care, 59 were retained in care, and 65 were virally suppressed [2]. Moreover, in 2018, according to the CDC, fewer Latinos disproportionately were virally suppressed compared to Whites (63.7% versus 70.7%, respectively) [3]. Furthermore, among Latinos who tested HIV-positive in 2014, only 64.0% were referred to risk-reduction services, based on additional CDC data [4]. Thus, there is an urgency to increase awareness of HIV, knowledge of one’s HIV status and engagement in medical care for Latinos found to be HIV-positive, especially men who have sex with men (MSM), the largest category [2, 5–7]. Linkages should also be made to HIV prevention services, as they can help to reduce the spread of HIV.

HIV prevention and care interventions have been developed for Latino MSM. Three are identified in the CDC *Compendium of Evidence-Based Interventions and Best Practices for HIV Prevention*: Hola en Grupos (Hello in Groups) [8]. No Excuses/Sin Buscar Excusas [9] and STYLE (Strength through Livin’ Empowered) [10]. The first two are Latino MSM-specific group HIV prevention interventions that focus on increasing HIV testing and condom use. They provide HIV-related information, teach safer sex skills, incorporate Latino cultural values and offer HIV-related services. The third intervention was developed for HIV-positive Latino and African American young MSM (17–24 years) and involves both individual and group sessions with a focus on linking individuals to HIV medical care and improving retention in care.

Other HIV prevention interventions for Latino MSM designed to increase HIV testing and reduce unprotected sex are described in a systematic review published in 2018 [11]. This review concluded that, though successful outcomes were obtained in some of the studies reviewed, there was an overall lack of inclusion of significant Latino cultural values among them. It called for the development of more interventions for Latinos, given the high burden of HIV experienced by them.

Research suggests that existing social and sexual networking mobile applications (“apps”) provide an effective avenue for recruiting men who have sex with men (MSM) for HIV-related interventions because of MSM’s frequent use of such apps [12–15]. Also, many MSM who use apps engage in high-risk sexual activities, making them an important group to target for HIV prevention [16]. Additionally, the use of these types of apps has been associated with having a sexually transmitted disease among MSM [17].

Most research conducted with MSM through apps has focused on obtaining survey data related to HIV risk behaviors and related topics [18–30]. Other studies have used apps to recruit individuals for HIV prevention interventions [15, 31–35]. These studies have examined the use of apps related to HIV and STD testing referrals [15], HIV vaccines [31], rectal microbicides [32] and HIV self-tests [33–35]. However, recent research suggests that existing apps may also be used to promote users’ involvement in HIV prevention activities, such as HIV testing [36].

Research on apps use by MSM is conducted worldwide. Examples of this include studies from Australia [37], Brazil [38, 39]; Canada [40], China [41–47], Ecuador [48, 49], England [50], Japan [51], the Netherlands [52], the Philippines [53] and Spain [54, 55]. Most of these studies explored topics such as users’ experiences connecting with others through apps, engaging in high-risk behaviors, and using apps for obtaining sexual health and HIV prevention information, as well as the opportunities provided for promoting HIV testing and linkage to HIV treatment via apps to users. Two studies focused on using existing apps for linking MSM to specific HIV testing sites, with one in Australia [56] and the other in Taiwan [57].

To our knowledge, no research has been reported which uses apps for connecting Latino MSM to HIV testing at a community based organization. However, such organizations play an essential role in identifying HIV-positive individuals. Thus, such research is crucial.

*Proyecto Protégete* (*Project Protect Yourself*) was a research study that developed an HIV prevention intervention in Los Angeles County, California, USA, to increase HIV testing and medical and prevention services utilization among English- and Spanish-speaking Latino MSM. It was a community-based HIV prevention research project that developed as a full partnership between a university and a Latino community agency serving the MSM population. The academic staff consisted of two investigators (one with a PhD in Urban Planning and Post-Doctoral training in Public Health and the other with a PhD in Biostatistics/Epidemiology); the agency staff consisted of the Director of Research and Evaluation (with a PhD in Social Welfare), a master’s level statistician, a Project Coordinator and two Project Interviewers. The PhD-level staff of both institutions collaborated on all aspects of the study: conceptualization of the research idea, methodology, funding acquisition, the project’s implementation, the interpretation of the study results and the writing of articles for academic journals.

The intervention, *Proyecto Protégete*, was not an app itself. It consisted of using existing apps targeted to MSM and financial remuneration. A website was developed for potential study participants to enroll into the program through their use of existing apps for connecting with other men. Further description of *Proyecto Protégete* is found below in Methods.

The primary aim of this study was to assess whether *Proyecto Protégete* was able to successfully recruit, through social and sexual networking mobile applications, Latino MSM involved in high-risk sexual activities. A secondary aim was to evaluate the project’s ability to promote HIV testing and linkage to HIV medical care and prevention services among this population by comparing it to the local standard of care. The local standard of care was defined as being the interventions conducted by the targeted HIV testing programs funded by the County Department of Public Health and implemented by many community based agencies across the County. This included providing HIV testing, linking HIV-positive people to HIV medical care and connecting individuals to HIV prevention services. The typical ways of initiating standard care included HIV testing conducted at storefronts and the use of HIV mobile testing vans for community outreach at gay bars, community festivals, health fairs and Gay Pride events.

*Proyecto Protégete* was developed because there was a concern that some Latino MSM who engaged in behaviors at high risk for HIV may not have been accessing the services provided by the local standard of care and possibly could be reached better through an intervention using apps. This article reports the development of the intervention, its implementation, results, challenges and recommendations for further research that may build on its experiences to encourage more Latino MSM to test for HIV and engage in medical and prevention services.

## Methods

### Developing *Proyecto Protégete*

For the purpose of developing *Proyecto Protégete*, eight focus groups were conducted between September 2014 and January 2015 in two of the Los Angeles City offices (Hollywood and East Los Angeles) of the Agency. Four focus groups were held for HIV-positive Latino MSM and four for HIV-negative Latino MSM. Eligibility criteria consisted of being a Latino man, 18 years of age or older and having sex with men. Recruitment occurred through the use of a promotional flier that was distributed at the different offices in Los Angeles County of the Agency (four of which were located in the City of Los Angeles) and through an ad published in a magazine directed to Latino MSM. Each focus group member received a gift card in the amount of $40.

Half of the groups were in Spanish and the other half in English. There was a total of 52 participants (29 Spanish- and 23 English-speakers; 24 HIV-negative and 28 HIV-positive). The focus groups included topics such as the identification of the social and sexual networking mobile apps used by the participants, the preferred times and days of the week for using apps, the specific apps that should be used by *Proyecto Protégete* to recruit Latino MSM for HIV testing, culturally appropriate approaches and techniques to incorporate for engaging Latino MSM into HIV testing through apps, the types of messaging that should be used and the specific financial amounts to offer as remuneration for getting tested for HIV and following through with referrals offered to HIV prevention services and medical care (the latter for those found to be HIV-positive).

The focus groups were recorded, and transcripts were made of the recordings. Two project staff members (one with a Doctorate in Public Health and the other a doctoral student in Social Psychology) analyzed the contents of the transcripts. This was done in a systematic and inductive manner drawing from grounded theory and using thematic analysis, which included becoming familiar with the transcripts, organizing the data by developing codes, combining the codes to develop themes and determining the completeness of the themes [58]. The two staff determined the proper interpretation of the codes and themes. Any disagreement between them was resolved through discussions until they reached consensus on their interpretations.

Based on the information collected from the focus groups, contact was made with the specific app companies chosen for use by the project. Discussions occurred regarding the particulars of the apps and how they could be used to recruit Latino MSM. This included the use of features such as pop-up ads, banner ads and direct recruitment. One company made the use of their app conditional on an agreement to never use their name in any future publication that associated their app with unsafe sexual activities by their users. This restriction was accepted, and a decision was made by the research team not to mention any app’s name in publications coming from the project. Seven apps were selected to recruit Latino MSM.

Additionally, a website developer was contracted to design the project’s website and its various pages (e.g., the Welcome page, the Screener page, the Eligibility page). The developer helped to determine the method for providing numerical codes, which served as unique identifiers for participants’ use in the project. An internal counter was developed for the website that was used for tabulating the number of submissions (i.e., the number of completed screeners submitted by potential study participants), the number of eligible participants (i.e., those who met all the eligibility criteria of the study), and the total number of submissions at any one time. The developer also created a section where the required information from the participants was available for viewing (e.g., age, which app was used to connect with the project). A link to the project’s website was incorporated into each of an app’s features described above (e.g., pop-up ads, banner ads). Individuals recruited for the study through the existing apps subsequently connected with *Proyecto Protégete* as described further below. The recruitment period for the intervention was from December 18, 2015 to April 22, 2017.

### Inclusion criteria

The study samples were *Proyecto Protégete*, the HIV testing program of the agency in which *Proyecto Protégete* was located [henceforth referred to as “Agency”] and the County Department of Public Health-funded HIV testing programs [henceforth referred to as “County”]. The HIV testing program of the Agency was one of the County-funded programs. The inclusion criteria for participants in all three samples consisted of being Latino, MSM, 18 years of age or older, and English- or Spanish-speaking.

### Use of apps to recruit participants

Various modes of advertisement were used in the apps, including pop-up messages and banner ads, to recruit participants. These advertisements were done in Spanish, English and “Spanglish” (a combination of both languages, as used sometimes by Latinos born in the United States). All advertisements included the project’s name, along with the project’s logo, which depicted the image of a young Latino man embracing himself (consistent with the idea reflected in the project’s name of the need to protect oneself from HIV infection). Examples of messages included the following: “Vatos chulos know their status–Get tested today!” (“Vatos chulos,” used by Mexican American men, translates to “cute dudes” in English.), “Live in the moment! Get tested today and receive a gift card!” and “Top or Bottom, HIV doesn’t discriminate–Get tested and receive a gift card.” The advertisement image and messages were consistent with the recommendation to incorporate provocative images and phrases that catch the attention of MSM apps users when attempting to engage them with HIV-related services [59].

When an individual clicked on the project’s advertisement link in an app, he was redirected to the project’s website screener page. There he answered questions to determine his eligibility for the study. If not eligible, he was directed to a page containing information about the Agency’s HIV testing and prevention programs. If eligible, he was directed to a page where he received a computer-generated unique code. He also responded to three of several security questions so that his code could be retrieved if forgotten. In addition, he was given information about how to get free HIV testing and financial remuneration for doing so, as part of his participation in the project.

None of typical ways of initiating standard care described previously (e.g., community outreach at gay bars, health fairs) were used by *Proyecto Protégete* for recruiting participants. Its project staff conducted its outreach solely through the apps identified for use by the project.

### HIV testing

When going to one of the three project locations at three of the six offices of the Agency in which *Proyecto Protégete* was housed (Hollywood, East Los Angeles and Pomona), an individual was asked for his code to identify him as a participant of *Proyecto Protégete*. Once his security code was matched, he was administered a study consent form which included permission for the project staff to ask an HIV counselor for his HIV test result following testing. Upon signing the consent form, he was considered enrolled in the project. A brief survey was then administered to gather information on demographics, sexual behaviors and drug use. The participant also filled out a locator form to enable the project staff to contact him if necessary to determine whether or not he had followed up with referrals to an HIV prevention program and HIV medical care (if found to be HIV-positive).

Once all of this was completed, the project staff took the individual to an HIV counselor for HIV testing. If he received a negative test result, he was able to reenroll in the project and get tested again every three months after his last enrollment. A participant reenrolling needed to go through all of the same steps, similar to his initial enrollment, every time he reenrolled.

### Referrals to HIV medical care and prevention services

Participants were referred to HIV medical care and prevention services, as needed, by the HIV counselor. All those who were HIV-positive were referred to one of the Agency’s HIV care peer navigators who would then connect them to an HIV medical provider.

All participants were provided with a list of HIV prevention programs. The HIV prevention programs to which participants were referred were internal to the Agency and directed to Latino MSM. Individuals were allowed to choose which program to attend among the twelve offered to them. These included programs for HIV-positive MSM and HIV-negative MSM. The services were provided in English or Spanish and were offered individually or in groups. Topics addressed in these programs included risk reduction strategies, safer sex negotiation, condom use, HIV transmission, alcohol and drug abuse, HIV stigma, mental health, support groups, and coming out to family and friends as a gay Latino man.

### Financial remuneration

Financial remuneration was used to motivate participants to access HIV testing, medical care and prevention services. Providing monetary incentives to an individual, subject to the completion of a behavioral goal, has been shown to have generally promising effects in programs, such as those focused on HIV testing [60]. The financial incentive for taking an HIV test was provided at the conclusion of the enrollment session. In order to receive an incentive for a medical referral, participants needed to have their attendance at their first medical appointment confirmed on a form signed by a medical clinic staff and subsequently submitted to the research team.

For incentives involving referrals to the Agency’s HIV prevention programs, the same protocol was followed as described above. In this case, the Agency staff member conducting the specific prevention program which the participant had chosen to attend needed to sign the form. The participant subsequently had to submit the form to the *Proyecto Protégete* staff within five days of his attendance in the prevention program. This provided documentation that he had kept his appointment.

Participants received incentives of $30 for an HIV test, $25 for attending the first session of an HIV prevention program, and $20 for attending the first HIV medical care appointment. To receive an incentive for HIV medical care, a participant needed to complete his first medical appointment within 90 days of receiving his HIV diagnosis. To receive an incentive for attending an HIV prevention program, participants needed to complete the referral within 30 days. Incentives were paid only to the *Proyecto Protégete* participants. They were not paid to the men in the two other study samples since payments were not part of the “standard of care”.

### Confirmation of new HIV diagnoses

The HIV test results from the project were subsequently submitted to the County Department of Public Health for verification regarding whether the tests were new diagnoses or already in their system (with the latter suggestive of a participant’s involvement in the study though likely already aware that he was HIV-positive).

### Data obtained for comparison purposes

Data regarding the number of HIV tests, confirmed new positives and linkages to medical care within 30 days and 90 days for the Agency, the County and *Proyecto Protégete* were obtained from the County Department of Public Health. Data regarding *Proyecto Protégete* participants successfully linked to HIV prevention services and the individuals linked to HIV prevention services by the HIV testing program of the Agency were obtained from the HIV Testing Department of the Agency. Both the *Proyecto Protégete* participants and the Agency’s clients were referred to the same HIV prevention programs at the Agency.

### Ethics approval

This study was approved by the Los Angeles County Public Health and Health Services Institutional Review Board and the University of California, Los Angeles Institutional Review Board. Signed informed consent was obtained from each individual prior to his participation in the study.

### Statistical analyses

Descriptive statistics were obtained for all the measures of interest. In addition, preliminary exploratory comparisons were made between *Proyecto Protégete* and the local standard of care regarding the rates of HIV-positive Latino MSM identified through HIV testing, linked to HIV medical care and linked to HIV prevention services.

For the first comparison, *Proyecto Protégete*’s HIV-positive testing rate was compared to the HIV-positive testing rates of the Agency and the County. For the second comparison, *Proyecto Protégete*’s rate of linked referrals to HIV medical care was compared to the Agency’s and the County’s rates for both 30 days and 90 days following an HIV diagnosis. For the third comparison, *Proyecto Protégete*’s rate of linked referrals to HIV prevention services within 30 days of receiving a referral was compared to the Agency’s rate for a similar time period. (Similar information was not available for the County.)

The number of tests and not the number of individuals was used in the analyses to examine the first comparison. This was because the County used unique tests and not unique individuals in their estimations; thus, the same estimates were used in the present analyses to make the results comparable. For the second and third comparisons, the number of individuals was used for the analyses.

Due to the relatively small counts available for analysis, the Fisher’s Exact Test was used for the comparisons, utilizing SPSS Statistics for Windows (Version 19.0, Armonk, NY: IBM Corp.) In all of the analyses, a one-sided test rather than a two-sided test was used to gain additional power because it was expected that *Proyecto Protégete* would perform better in identifying HIV-positive cases and linking individuals to HIV medical care and prevention services compared to the Agency and the County. Please note that the numbers reported for the Agency and for the County always exclude the data for the participants of *Proyecto Protégete*. Also, the date range under examination for the Agency and the County was the same as the recruitment period for *Proyecto Protégete* identified previously.

## Results

### Project submissions, eligible individuals and enrollees

There were 9,573 submissions (individuals who completed the online screener for the project through an app). Of these submissions, 4,657 were eligible to participate in the study. From these, 359 (i.e., 7.7% of those who were screened as eligible) enrolled in the project.

### Demographic and behavioral characteristics of *Proyecto Protégete* participants

Summarized data from Table 1 reveal that the largest age group among the participants was between 26 to 35 years and the second largest between 18 to 25 years of age. Most were born in the US, followed by Mexico. A fifth had a bachelor’s degree, and almost a quarter completed only the 12^th^ grade. Most worked either full- or part-time. The previous year’s income category of 0 to $15,000 had the largest number of participants, approximately 43%. Seventy seven percent of the sample consisted of United States citizens, and 12% were undocumented. Forty one percent reported two to five sexual partners in the previous twelve months, 26% six to ten and 26% more than ten. Seventy nine percent reported anal sex without a condom with a man in the previous twelve months. Thirteen percent reported anal sex under the influence of methamphetamine with another male during this same period, and over half reported anal sex under the influence of alcohol. Methamphetamine use in the previous twelve months was reported by 13% and cocaine use by 14%.

**Table 1:** Demographics, sexual behavior and alcohol/drug use among *Proyecto Protégete* participants (N = 359).

Variable	n	%
Age		
18–25	113	31.5
26–35	147	40.9
36–45	68	18.9
46–55	28	7.8
56–65	3	0.8
Country of Birth^a^		
Other	33	9.2
Mexico	74	20.7
United States	250	70.0
Education		
Less than Grades 12	21	5.9
Grade 12	81	22.6
GED	3	0.8
Technical Degree	12	3.3
Some College	131	36.5
Associate Degree	28	7.8
Bachelor Degree	72	20.1
Other	11	3.1
Employment		
Working full-time	161	44.8
Working part-time	98	27.3
Unemployed	77	21.4
Disabled	11	3.1
Retired	1	0.3
Other	11	3.1
Income in Previous Year before Taxes^b^		
0-$15,000	152	42.7
$15,001-$30,000	111	31.2
More than $30,000	93	26.1
Residency Status^c^		
U.S. citizen	276	77.1
Legal resident	23	6.4
Undocumented	41	11.5
Other	18	5.0
Number of Sexual Partners in Previous 12 Months^b^		
0	3	0.8
1	20	5.6
2–5	146	41.0
6–10	93	26.1
More than 10	94	26.4
Anal Sex without a Condom with a Man in Previous 12 Months^a^	283	79.3
Anal Sex with a Man in Previous 12 Months While Under the Influence of Methamphetamine^a^	47	13.2
Anal Sex with a Man in Previous 12 Months While Under the Influence of Alcohol^a^	183	51.3
Drug Use in Previous 12 Months		
Methamphetamine	48	13.4
Cocaine	50	13.9

^a^ n = 357;

^b^ n = 356;

^c^ n = 358

### HIV testing outcomes

For the 359 unique participants enrolled in the study, the total number of HIV tests conducted for *Proyecto Protégete* was 428. A total of 50 individuals tested for HIV more than once: 36 people took one additional test; ten people took two additional tests; three people took three additional tests, and one person took four additional tests.

### Comparisons between *Proyecto Protégete* and the local standard of care

Table 2 provides information on the numbers of HIV tests, confirmed new HIV-positive tests, and individuals linked to medical care within 30 days and 90 days for the County, the Agency and *Proyecto Protégete*.

**Table 2:** HIV tests, confirmed new HIV-positive tests, and individuals linked to medical care within 30 and 90 days among Latino MSM, 18 years or older at time of test for the county, the agency and *Proyecto Protégete*.

HIV Testing Program	HIV Tests	Confirmed New HIV-Positive Tests	Individuals Linked to Medical Care within 30 Days	Individuals Linked to Medical Care within 90 Days
County^a^	17,721	194 (1.09%)	150 (77.3%)	167 (86.1%)
Agency	2,158	27 (1.25%)	22 (81.5%)	25 (92.6%)
*Proyecto Protégete* ^b^	428	7 (1.71%^c^)	5 (71.4%)	7 (100.0%)

^a^ This refers to the County’s directly-funded targeted testing programs. It does not represent the general population of the County.

^b^ Records identified as being of individuals in *Proyecto Protégete* are excluded from those of the County and the Agency.

^c^ The number of tests that was used for the denominator was 410 (please see text).

Originally, 25 HIV-positive test results were found for *Proyecto Protégete*; however, only 22 of those matched the records in the County Department of Public Health database (possible reasons for these two numbers not matching include the tests being done for a contract not tracked by the County in the same manner that it does for its own directly-funded targeted HIV testing programs or possible recording errors). Of the 22 HIV-positive results from *Proyecto Protégete*, only 7 (32%) of them turned out to be newly diagnosed, as confirmed by the County Department of Public Health. Thus, *Proyecto Protégete’*s positivity rate was 7/(428–18) = 1.71%, once the 18 tests not confirmed as true newly diagnosed positive results (the 3 HIV-positive tests that did not match the records in the County database and the 15 HIV-positive tests of individuals likely already aware of their HIV-positive status before they started *Proyecto Protégete*) were excluded from the denominator. The comparable HIV-positivity rate for the Agency was 27/2,158 = 1.25% and for the County, 194/17,721 = 1.09%. When *Proyecto Protégete*’s HIV positivity rate was compared to that of the HIV testing program at the Agency, the result was not significant (*p* = .293). When *Proyecto Protégete*’s HIV positivity rate was compared to that of the County, the result was also not significant (*p* = .172).

When examining the rates of those confirmed as new HIV-positives and linked to medical care within 30 days, the following rates were obtained: for *Proyecto Protégete*, 5/7 = 71.4%; for the HIV testing program at the Agency, 22/27 = 81.5%; and for the HIV testing programs of the County, 150/194 = 77.3%. *Proyecto Protégete*’s rate of linkage to medical care within 30 days did not significantly differ from that of the HIV testing program at the Agency (*p* = .450) or from that of the County (*p* = .503). The rates of those confirmed as new HIV-positives and linked to care within 90 days were then examined. The rate for *Proyecto Protégete*, 7/7 = 100.0% did not significantly differ from the rate for the HIV testing program at the Agency, 25/27 = 92.6% (*p* = .626) or from the rate for the HIV testing programs of the County, 167/194 = 86.1% (*p* = .358).

All of the 359 individuals enrolled in *Proyecto Protégete* were provided with referrals to one of the Agency’s prevention programs. Of these, 70 (19.5%) confirmed their attendance. *Proyecto Protégete*’s rate of linked referrals to HIV prevention services was then compared to the Agency’s HIV testing program’s rate of linked referrals to HIV prevention services for a similar time period. The rate for the Agency was 17/204 = 8.3%. *Proyecto Protégete* had a significantly higher rate of linked referrals to HIV prevention services than did the Agency’s HIV testing program (*p* < .001).

## Discussion

The approach utilized by *Proyecto Protégete* for recruiting Latino MSM involved in high-risk sexual activities with other men in Los Angeles County, California, USA, for HIV testing through the use of existing apps and financial remuneration was found to be feasible and successful, thereby accomplishing the primary aim of the study. As noted previously, 79% of the participants reported having anal sex without a condom with a man in the previous twelve months. Also, over half reported anal sex under the influence of alcohol with another male, and 13% reported anal sex under the influence of methamphetamine. Thus, a large percentage of participants was at high risk for HIV infection and in need of the services provided by *Proyecto Protégete*. Additionally, over 70% of the participants were between the ages of 18 and 35 years, a range that includes those with the highest rates of HIV infection among Latinos [1]. Studies wanting to reach Latino MSM at high risk for HIV infection should consider using existing apps in their interventions.

Additionally, *Proyecto Protégete* had a significantly higher rate of successfully linked referrals to HIV prevention programs than did the Agency’s HIV testing program, thus partially accomplishing the secondary aim of the study. Prevention programs help to increase awareness of HIV and knowledge of one’s own HIV status, both of which are fundamental in preventing new HIV infections [7]. Interventions like *Proyecto Protégete* that incorporate linguistically and culturally sensitive approaches to facilitate HIV testing and engagement to medical care as well as linkages to prevention programs have a significant role to play, as they may help to decrease HIV spread among Latinos [4].

It is likely that the monetary incentives paid to the *Proyecto Protégete* participants may have been the main driver of the difference in completed referral rates to HIV prevention programs between the program and the Agency, whose participants received no financial incentives. This possibility is reinforced by the fact that the lowest income category in the study was reported by the largest number of participants, who may have been in need of the monetary incentive. Thus, it is possible that no difference may have been found between the two groups if financial incentives had been available through the Agency’s usual work.

Although *Proyecto Protégete* was demonstrated to be more effective, with a higher HIV-positivity rate, than the programs of the Agency and the County, statistical significance was not reached with either comparison. This may have been due to the sample size of *Proyecto Protégete*, with subsequent limited statistical power. Future research should build on the present study by using larger samples.

Alternately, it is also possible that the sample collected for *Proyecto Protégete* was not more likely to be HIV-positive than those of the Agency and the County. Despite the fact that MSM who use apps have been found to engage in high-risk sexual activities [16] and the use of apps being associated with having a sexually transmitted disease [17], no differences in the HIV-positivity rates in the two comparisons were discovered. More needs to be known about the populations reflected in the samples of this study. Future studies should examine whether or not differences in the rates of behaviors associated with a risk of HIV infection exist between Latino MSM who connect with HIV testing services through apps compared to those who do so through the local standard of care.

Despite the lack of statistical differences in the HIV-positivity rates between *Proyecto Protégete* and the other two samples, it is possible that there were differences in other areas between the newly diagnosed HIV-positive participants of *Proyecto Protégete* and those of the two other programs that would have been valuable to discover. The County Department of Public Health had indicated that they would only provide the totals for the variables of interest regarding the three programs, as reflected in Table 2. It added that it would not provide information regarding which of the tests submitted to them by *Proyecto Protégete* were of actual newly diagnosed HIV-positive individuals. Thus, the identities of the participants who met this criterion remained unknown to the *Proyecto Protégete* staff. As a result, comparisons of the demographics of the newly diagnosed HIV-positive individuals of *Proyecto Protégete* to those of the Agency and the County were not possible. In addition, comprehensive information on the HIV testing histories of *Proyecto Protégete* participants was not collected. This information would have been valuable for assessing the experiences of these individuals with also accessing HIV testing services through the local standard of care. Future studies should investigate how the demographics and HIV testing histories of Latino MSM who engage in HIV testing through apps differ from those who connect to HIV testing services solely through the local standard of care.

Examining the economic costs of *Proyecto Protégete*, the Agency and the County was not an objective of the study. Nevertheless, an economic comparison of HIV testing programs would be of value. This should include not only the economic costs of the programs but also their social costs. Cost-effectiveness analysis, which provides the opportunity to incorporate social costs, can be used for this purpose [61–65]. Two social costs to consider may be quality-adjusted life years (QALY) [61, 66, 67] and averted HIV infections [66–68]. Cost-effectiveness analysis is recommended because research has found that the inclusion or exclusion of social costs can affect the outcomes of some analyses, as demonstrated in systematic reviews of economic evaluations of physical [63] and mental diseases [61].

Two HIV testing intervention studies conducted in other countries showed some similarity to *Proyecto Protégete*. The first study, conducted in Sydney, Australia, between December 2013 and May 2015, sought to determine the effects of the placement of pop-up and banner ads on Grindr, a popular app used by MSM, on the average monthly rate of HIV tests among MSM at a particular sexual health clinic [56]. Such advertising was found to result in a 43.6% increase in the average monthly rate of HIV tests among this population.

The second study, conducted in Taipei and New Taipei City in Taiwan, between May and November 2018, sought to compare two mobile HIV testing recruitment approaches with MSM: recruitment through a website and recruitment through social networking platforms [57]. The website approach involved promoting HIV testing through a public website of a hospital and transmitting that information to a location frequented by MSM. The social networking platforms approach used both existing apps and a Facebook page to promote information about HIV testing. The social networking approach resulted in a greater likelihood of reaching MSM with higher HIV risk-taking behaviors compared to those recruited through a website; additionally, the social networking approach had HIV-positivity rates that were three times higher than the website approach.

As was the case with *Proyecto Protégete*, both of these studies used existing apps to promote HIV testing by MSM for specific programs. Additionally, they reported success in increasing HIV testing among MSM, reaching MSM with high HIV risk-taking behaviors and identifying high HIV-positivity rates. These findings support the value of using apps for promoting HIV testing at specific venues, such as health clinics, and through mobile HIV testing programs.

Replication of *Proyecto Protégete* in full or in part in other countries will need to consider modifications tailored to the specific conditions of those locations. These will require taking into account issues such as the program’s feasibility, cost, cultural compatibility, challenges and impediments in new settings. Together, these efforts can contribute to a greater use of apps as health promotion vehicles to lower the incidence of HIV among MSM globally.

There were some challenges that prevented *Proyecto Protégete* from being more successful. One was the low percentage of individuals (7.7%) who enrolled in the project after being screened as eligible for the study. Despite the anticipation that individuals who had expressed an interest in the project by taking the eligibility screener would actually enroll, this did not prove to be as successful as expected. However, other HIV prevention intervention studies in the United States using apps to recruit participants generally have also had low rates of enrollment with, for example, 2.8% [33] and 7.6% [35] for studies of HIV self-tests, 6.9% for an HIV prevention vaccine trial [31] and 1.7% for a rectal microbicide study [32]. Thus, *Proyecto Protégete’s* rate of enrollment matched others found in the literature. Nonetheless, research using apps should identify ways to increase participation among Latino MSM at risk for HIV, such as having more locations where the study is offered, assisting with transportation and increasing financial remuneration.

Another challenge was the low number of participants in *Proyecto Protégete* who successfully linked to an HIV prevention program within 30 days (19.5%), although, as noted above, the rate for *Proyecto Protégete* was significantly higher than the rate for the Agency. It had been hoped that the modest financial remuneration would encourage greater interest in the prevention programs by the participants, but it did not. Future studies should focus on ways to increase participation among Latino MSM in HIV prevention programs following an HIV test, as there is evidence of their potential effectiveness in reducing risk behaviors among Latinos [4, 11].

A limitation of the study that was not discovered until the final results from the County Department of Public Health were received was the large number of individuals in *Proyecto Protégete* who already may have known their HIV-positive status at the time that they enrolled in the project. Of the 22 HIV-positive test results for *Proyecto Protégete*, only 7 (32%) turned out to be newly diagnosed. Thus, 15 test results (68%) were of individuals likely already aware of their HIV-positive diagnosis. This issue was addressed by including only those 7 newly diagnosed HIV-positive test results in the determination of *Proyecto Protégete’*s HIV-positivity rate.

Regarding this situation of the HIV-positive tests which had to be removed from the analysis, the study screener did not include a question asking if the potential participant had previously been diagnosed with HIV. Nonetheless, *Proyecto Protégete* had a study protocol in place that could have potentially identified those individuals who may have already been aware of their HIV-positive diagnosis prior to their enrollment in the study. The data collection form of the HIV counselor who administered the HIV test to the participant specifically included a question that asked if the person had tested for HIV in the past and, if so, what their last HIV test result was. Given that a participant had signed a project consent form which included his permission for the *Proyecto Protégete* project staff to ask the HIV counselor for the results of the person’s HIV test, the counselor would have revealed to the project staff that someone had already previously been diagnosed with HIV, if the individual had disclosed this information to them. It cannot be known with certainty the reasons that may have motivated the participation by individuals who may have already been aware of their HIV-positive status (such as intentional deception in order to receive the financial remuneration offered by the study). Studies should include an explicit question of previous HIV testing in their screeners and incorporate protocols that prevent individuals who do not qualify from participating in a study. Nevertheless, a strength of this study was its ability to cross reference, through information obtained from the County Department of Public Health, whether or not participants had previously already been given a diagnosis of being HIV-positive. Those who had were subsequently removed from the statistical analyses conducted.

Another limitation was the fact that this study used data from three different sources: the County, the Agency and *Proyecto Protégete*. There are always potential problems in making comparisons when using multiple data sources. These can include differences among the sources in the accuracy and completeness of the data collected and reported, resulting in inaccurate coding, poorly conducted data collection and data entry [69]. It is possible, then, that this may have occurred in our study, especially since the County sample consisted of data collected from multiple County-funded HIV testing programs located in different agencies spread throughout the County.

## Conclusion

*Proyecto Protégete* was successful in recruiting its desired target population, Latino MSM involved in behaviors that put them at high risk for HIV infection and thus in need of the services of the project. In addition, all of the individuals in *Proyecto Protégete* who were confirmed as newly HIV-positive were successfully linked to medical care. Furthermore, *Proyecto Protégete* was found to have a higher rate of linked referrals to HIV prevention services compared to the Agency’s HIV testing program. However, no statistical differences were found when *Proyecto Protégete* ‘s HIV positivity rate was compared to that of the Agency or to that of the County. Similarly, *Proyecto Protégete*’s rates of linkage to medical care, both within 30 days and 90 days, did not significantly differ from those of the Agency or the County. Nonetheless, future studies can build on the experiences of *Proyecto Protégete* when using apps for promoting HIV services among Latino MSM involved in HIV-related high-risk behaviors.

## Supporting information

S1 File English survey. (DOC)

S2 File Spanish survey. (DOCX)

S3 File Dataset for Table 1. (XLS)

S4 File Note on Table 2 data. (DOCX)
