# Supplementary material for: Using social and sexual networking mobile applications to promote HIV testing, medical care and prevention services among Latino men who have sex with men in Los Angeles County, California, USA
Source: PLoS One. 2022 May 13;17(5):e0268406. doi: 10.1371/journal.pone.0268406 (PMC9106153; doi:10.1371/journal.pone.0268406)
Supplement: S1 File — (DOC) [file pone.0268406.s001.doc]

**Brief Survey for Intervention Phase**

**(CHRP: *Using Social Media with Latino MSM for HIV Testing and Linkage*)**

Interviewer: __________________

Participant ID #: ______________

Date of Interview: _____________

**I will begin by asking you some questions about your use of social networking sites.**

1. "Do you use apps that are for hooking up with or dating men?" _____

**INTERVIEWER INSTRUCTION: IF THE PARTICIPANT ANSWERED, “NO,” SKIP TO QUESTION #7.**

1. How many days a week do you use social networking apps/sites? ___________
2. What days of the week do you use them?

**INTERVIEWER INSTRUCTION: CHECK AS MANY AS APPLY**

___ Monday

___ Tuesday

___ Wednesday

___ Thursday

___ Friday

___ Saturday

___ Sunday

1. At what times of the day do you use them?

**INTERVIEWER INSTRUCTION: CHECK AS MANY AS APPLY**

___ Early mornings (midnight 12:00 a.m. to 6:00 a.m.)

___ Mornings (6:00 a.m. to 12:00 p.m.)

___ Afternoons (12:00 p.m. to 6:00 p.m.)

___ Evenings (6:00 p.m. to 10:00 p.m.)

___ Late nights (10:00 p.m. to midnight 12:00 a.m.)

1. What is the primary reason you use these apps/sites?

**INTERVIEWER INSTRUCTION: CHECK ONLY ONE OF THE FOLLOWING**

___ Sex (hook-up)

___ Party and Play (sex and drugs)

___ Sex work

___ Escort

___ Dating

___ Loneliness

___ Boredom

___ Entertainment

___ To meet someone

___ Friendship (to keep in touch, to see who is there)

___ To meet someone to get a place to stay

___ Other: _________________________ **(INTERVIEWER INSTRUCTION: IF CHECKED, PLEASE**

**SPECIFY)**

1. For what other reasons do you use these apps/sites?

**INTERVIEWER INSTRUCTION: CHECK AS MANY AS APPLY**

___ Sex (hook-up)

___ Party and Play (sex and drugs)

___ Sex work

___ Escort

___ Dating

___ Loneliness

___ Boredom

___ Entertainment

___ To meet someone

___ Friendship (to keep in touch, to see who is there)

___ To meet someone to get a place to stay

___ Other: _________________________ **(INTERVIEWER INSTRUCTION: IF CHECKED, PLEASE**

**SPECIFY)**

**Now I will ask you some general questions about yourself.**

1. How old are you? __________________
2. What is the zip code where you currently live? ______________
3. What is the highest level of education that you completed?

A. Never attended school:__________

B. Grades 1 through 6:__________

C. Grades 7 or 8:__________

D. Grades 9 through 11:__________

E. Grade 12:__________

F. GED:__________

G. Technical degree:__________

H. Some college:__________

I. Associate degree:__________

J. Bachelor’s degree:__________

K. Other:__________

K1. What? ____________________________________

1. What is your current work situation?

_____Working full time
_____Working part-time

_____With a job but on sick leave

_____Unemployed and looking for work

_____Unemployed and not looking for work

_____Disabled and not working

_____Retired and not working

_____ Other (e.g. student, day laborer):

What? ____________________________________

1. Please tell me about how much income did you make in the last year before taxes?
2. $0-$5,000
3. $5,001-$10,000
4. $10,001-$15,000
5. $15,001-$20,000
6. ⁭$20,001-$25,000
7. ⁭$25,001-$30,000
8. $30,001- $35,000
9. $35,001- $40,000
10. $40,001- $45,000
11. $45,001- $50,000
12. More than $50,000

**Now I would like to ask you some questions about your drug use and sexual activity in the last 12 months. Answer in the best way you can and remember that your answers are completely confidential.**

1. In the PAST 12 MONTHS, have you
   1. Used Methamphetamine Yes____ No____
   2. Used Crack Yes____ No____
   3. Used Heroin Yes____ No____
   4. Used Cocaine Yes____ No____
   5. Injected any drug Yes____ No____
   6. Shared any injection equipment Yes____ No____
2. In the PAST 12 MONTHS, how many sexual partners did you have? _______

**INTERVIEWER INSTRUCTION: IF THE PARTICIPANT ANSWERED 0, SKIP TO THE STATEMENT BEFORE QUESTION #15.**

1. In the PAST 12 MONTHS, have you had vaginal or anal sex: (mark all that apply)
   1. With a condom Male____ Female____ TG____
   2. Without a condom Male____ Female____ TG____
   3. With a person who is IDU Male____ Female____ TG____
   4. With a person who is HIV-positive Male____ Female____ TG____
   5. Under the influence of Methamphetamine Male____ Female____ TG____
   6. Under the influence of Alcohol Male____ Female TG____

**Now we would like to ask you some questions about Pre-Exposure Prophylaxis or PrEP and Post- Exposure Prophylaxis or PEP.**

**PrEP**

1. Have you ever heard of Pre-Exposure Prophylaxis or PrEP for preventing HIV infection? PrEP involves taking an HIV medication EVERY DAY to help prevent HIV infection among people who are NOT infected with HIV. **INTERVIEWER DIRECTIONS: MAKE SURE TO READ TO THE CLIENT WHAT PrEP IS.**
   1. Yes
   2. No **(If no, skip to question # 18)**
2. Are you currently using PrEP.  That means a doctor prescribed a medication to take EVERY DAY to protect you from becoming infected HIV?

- 1. Yes **(If yes, skip to question # 19)**
  2. No

1. Do you know how to get PrEP?
   1. Yes
   2. No
2. How likely would you be to take PrEP to help prevent you from becoming infected with HIV?
   1. Extremely Likely
   2. Likely
   3. Indifferent
   4. Unlikely
   5. Very Unlikely

**PEP**

1. Have you heard of Post-Exposure Prophylaxis or PEP?  PEP involves taking HIV medications for one month after you think you may have been exposed to HIV to help prevent you from becoming infected with HIV.
   1. Yes
   2. No **(If no, skip to question number 22)**
2. Have you ever used PEP to prevent an HIV infection? That means a doctor prescribed HIV medications for a month after you think or know you were exposed to HIV.
   1. Yes **(If yes, skip to the statement before question number 23)**
   2. No
3. Do you know how to get PEP?
   1. Yes
   2. No
4. If you think you were exposed to HIV (a condom broke), how likely would you be  to take HIV medications for one month after you had been exposed  to help prevent you from becoming infected with HIV?
   1. Extremely Likely
   2. Likely
   3. Indifferent
   4. Unlikely
   5. Very Unlikely

**Finally, we would like to ask you some general questions about your background and residency status.**

1. From which Latin American country or countries did your parents or grandparents come? ____________________________________________
2. In what country were you born? __________________

**INTERVIEWER INSTRUCTION: SKIP TO QUESTION # 26 IF THEY WERE BORN IN THE U.S.**

1. How old were you when you first moved to the U.S.? _________________
2. What is your residency status in the United States?

**INTERVIEWER INSTRUCTION: READ ALL THE CATEGORIES**

a. U.S. citizen__________

b. Legal resident__________

c. Undocumented__________

d. Other__________

SPECIFY: ___________________________

Thank you for your participation.

INTERVIEWER CHECKPOINT:

PLEASE OBTAIN THE INDIVIDUAL’S HIV TEST RESULT FROM HIS HIV TESTER AND WRITE IT HERE:

HIV-NEGATIVE: __________

HIV-POSITIVE: __________
