# Supplementary material for: Using social and sexual networking mobile applications to promote HIV testing, medical care and prevention services among Latino men who have sex with men in Los Angeles County, California, USA
Source: PLoS One. 2022 May 13;17(5):e0268406. doi: 10.1371/journal.pone.0268406 (PMC9106153; doi:10.1371/journal.pone.0268406)
Supplement: S2 File — (DOCX) [file pone.0268406.s002.docx]

**Encuesta Breve para la Fase de Intervención**

**(CHRP: *Uso de Redes Sociales con Hombres Latinos que Tienen Sexo con Hombres para hacer la Prueba del VIH y Vínculo)***

Entrevistador: __________________

# de Identificación del Participante: ______________

Fecha de la Entrevista: _____________

**Voy a empezar por hacerle algunas preguntas acerca de su uso de redes sociales.**

1. "¿Usted utiliza aplicaciones que son para encuentros sexuales o para salir con hombres?" _____

**INSTRUCCION PARA EL ENTREVISTADOR: SI EL PARTICIPANTE RESPONDIO “NO,” PASE A LA PREGUNTA #7.**

1. ¿Cuantos días a la semana utiliza las redes/aplicaciones sociales? ___________
2. ¿Qué días de la semana usted las utiliza?

**INSTRUCCION PARA EL ENTREVISTADOR: MARQUE TODAS LAS QUE APLIQUEN**

___ Lunes

___ Martes

___ Miércoles

___ Jueves

___ Viernes

___ Sábado

___ Domingo

1. ¿A qué horas del día usted las utiliza?

**INSTRUCCION PARA EL ENTREVISTADOR: MARQUE TODAS LAS QUE APLIQUEN**

___ Por las madrugadas (de media noche 12:00 a.m. a 6:00 a.m.)

___ Por las mañanas (de 6:00 a.m. a 12:00 p.m.)

___ Por las tardes (de 12:00 p.m. a 6:00 p.m.)

___ Por las noches (de 6:00 p.m. a 10:00 p.m.)

___ Noches muy tardes (de 10:00 p.m. a media noche 12:00 a.m.)

1. ¿Cuál es la razón principal por la que usted las utiliza?

**INSTRUCCION PARA EL ENTREVISTADOR: MARQUE SOLAMENTE UNA DE LAS SIGUIENTES**

___ Sexo (encuentro sexual)

___ Party and Play (sexo y drogas)

___ Trabajo sexual

___ Escort

___ Noviar

___ Soledad

___ Aburrimiento

___ Entretenimiento

___ Para conocer a alguien

___ Amistad (para mantenerse en contacto, para ver quien está allí)

___ Para conocer a alguien para conseguir un lugar adonde quedarse (vivienda)

___ Otro: _________________________

**(INSTRUCCION PARA EL ENTREVISTADOR: SI MARCO AQUI, POR FAVOR ESPECIFIQUE)**

1. ¿Cuáles son otras razones por las que usted las utiliza?

**INSTRUCCION PARA EL ENTREVISTADOR: MARQUE TODAS LAS QUE APLIQUEN**

___ Sexo (encuentro sexual)

___ Party and Play (sexo y drogas)

___ Trabajo sexual

___ Escort

___ Noviar

___ Soledad

___ Aburrimiento

___ Entretenimiento

___ Para conocer a alguien

___ Amistad (para mantenerse en contacto, para ver quien está allí)

___ Para conocer a alguien para conseguir un lugar adonde quedarse (vivienda)

___ Otro: _________________________

**(INSTRUCCION PARA EL ENTREVISTADOR: SI MARCO AQUI, POR FAVOR ESPECIFIQUE)**

**Ahora le haré unas preguntas generales acerca de usted.**

1. ¿Qué edad tiene? __________________
2. ¿Cuál es el código postal de donde usted vive en este momento? ______________
3. ¿Cuál es el nivel más alto de educación que usted completo?

A. Nunca atendió una escuela: __________

B. Grados del 1^ro^ al 6^to^: __________

C. Grados del 7^mo^ o 8^vo^: __________

D. Grados del 9^no^ al 11^mo^: __________

E. Grado 12^mo^: __________

F. Desarrollo Educacional General (GED)

(Equivalente a la Escuela de Adultos): __________

G. Titulo técnico: __________

H. Un poco de universidad: __________

I. Carrera técnica (A.A.): __________

J. Licenciatura: __________

K. Otro: __________

K1. ¿Qué? ____________________________________

1. ¿Cuál es su situación actual de trabajo?

_____Trabajando tiempo completo
_____Trabajando de medio tiempo

_____Con trabajo pero enfermo

_____Desempleado pero busca trabajo

_____Desempleado y no busca trabajo

_____Discapacitado y no trabaja

_____Jubilado y no trabaja

_____Otro (p.ej., estudiante, jornalero):

¿Qué? ____________________________________

1. 14. Por favor, dígame cuanto hizo de ingresos el año pasado antes de pagar impuestos.
2. $0-$5,000
3. ⁭$5,001-$10,000
4. $10,001-$15,000
5. $15,001-$20,000
6. ⁭$20,001-$25,000
7. ⁭$25,001-$30,000
8. $30,001- $35,000
9. $35,001- $40,000
10. $40,001- $45,000
11. $45,001- $50,000
12. ⁭Más de $50,000

**Ahora me gustaría hacerle unas preguntas acerca de su uso de drogas y actividades sexuales en los últimos 12 meses. Responda de la mejor forma que usted pueda y acuérdese que sus respuestas son completamente confidenciales.**

1. En los ULTIMOS 12 MESES, usted ha
   1. Usado Metanfetamina Sí____ No____
   2. Usado Crack Sí ____ No____
   3. Usado Heroína Sí ____ No____
   4. Usado Cocaína Sí ____ No____
   5. Inyectado alguna droga Sí ____ No____
   6. Compartido algún equipo de inyección Sí ____ No____
2. ¿En los últimos 12 meses, cuantas parejas sexuales ha tenido usted? _______

**INSTRUCCION PARA EL ENTREVISTADOR: SI EL PARTICIPANTE RESPONDIO 0, PASE A LA FRASE ANTES DE LA PREGUNTA #15.**

1. En los ULTIMOS 12 MESES, usted ha tenido sexo vaginal o anal: (marque todas las que aplican)
   1. Con condón Hombre____ Mujer____ TG____
   2. Sin condón Hombre____ Mujer____ TG____
   3. Con una persona que inyecta drogas Hombre____ Mujer____ TG____
   4. Con una persona que es VIH-positivo Hombre____ Mujer____ TG____
   5. Bajo la influencia de la Metanfetamina Hombre____ Mujer____ TG____
   6. Bajo la influencia del alcohol Hombre____ Mujer____ TG____

**Ahora nos gustaría hacerle unas preguntas acerca de Profilaxis para la Pre-Exposición también conocido como PrEP y de Profilaxis para la Pos-Exposición conocido como PEP.**

**PrEP**

1. Alguna vez ha escuchado de PrEP (Profilaxis de Pre-Exposición) para prevenir la infección del VIH? PrEP involucra tomar medicamento para el VIH TODOS LOS DIAS para ayudar a prevenir la transmisión del VIH a gente que NO está infectada.
2. Si
3. No **(Si responde no, pase a la pregunta numero 18)**
4. ¿Está utilizando actualmente PrEP? Es decir que un doctor le ha recetado un medicamento para que la tome TODOS LOS DIAS para protegerlo de la infección del VIH.
5. Si **(Si responde si, pase a la pregunta numero 19)**
6. No
7. Sabe cómo conseguir PrEP?
8. Si
9. No
10. Cuál es la probabilidad de que tomaría PrEP para ayudarle a prevenir la infección del VIH?
11. Muy probable
12. Probable
13. Indiferente
14. Improbable
15. Muy improbable

**PEP**

1. Alguna vez ha escuchado de Profilaxis de Pos-Exposición, también conocido como PEP? PEP involucra tomar medicamentos para el VIH por un mes después de que usted piense que quizás se haya expuesto al VIH para ayudarle a prevenir la infección por el VIH.
2. Si
3. No **(Si responde no, pase a la pregunta numero 22)**
4. Alguna vez ha usado PEP para prevenir la infección del VIH? Es decir que un doctor le ha recetado medicamento para el VIH por todo un mes después de que usted piensa o sepa que ha sido expuesto al VIH.
5. Si **(Si responde si, pase a la frase antes de la pregunta numero 23)**
6. No
7. Sabe cómo conseguir PEP?
8. Si
9. No
10. Si piensa que ha sido expuesto al VIH (se rompió el condón), que tan probable seria que usted tomaría medicamento para el VIH por un mes después de haber sido expuesto al virus para ayudarle a prevenir la infección por el VIH?
11. Muy probable
12. Probable
13. Indiferente
14. Improbable
15. Muy improbable

**Finalmente me gustaría hacerle unas preguntas generales acerca de sus antecedentes y estatus de residencia.**

1. ¿De cuál país(es) de Latino América vinieron sus padres o abuelos?

______________________________________________

1. ¿En cuál paí**s** nació usted? __________________

**INSTRUCCION PARA EL ENTREVISTADOR: PASE A LA PREGUNTA #26 SI NACIO EN LOS E.E.U.U.**

1. ¿Qué edad tenía cuando se movió a los E.E.U.U. por primera vez?_________________
2. ¿Cuál es su estatus de residencia en los Estados Unidos?

**INSTRUCCION PARA EL ENTREVISTADOR: LEA TODAS LAS CATEGORIAS**

a. Ciudadano Estadounidense __________

b. Residente legal __________

c. Indocumentado __________

d. Otro __________

ESPECIFIQUE: ________________________________

Gracias por su participación.

INTERVIEWER CHECKPOINT:

PLEASE OBTAIN THE INDIVIDUAL’S HIV TEST RESULT FROM HIS HIV TESTER AND WRITE IT HERE:

HIV-NEGATIVE: __________

HIV-POSITIVE: __________
