# Supplementary material for: Using social and sexual networking mobile applications to promote HIV testing, medical care and prevention services among Latino men who have sex with men in Los Angeles County, California, USA
Source: PLoS One. 2022 May 13;17(5):e0268406. doi: 10.1371/journal.pone.0268406 (PMC9106153; doi:10.1371/journal.pone.0268406)
Supplement: S4 File — (DOCX) [file pone.0268406.s004.docx]

**Note on Table 2 Data**

No dataset has been submitted for the data presented in Table 2. With the exception of the number of HIV tests conducted for *Proyecto Protégete*, all of the other data were obtained from the County Department of Public Health. Subsequently, the necessary percentages were calculated with this information.
